# Oral Squamous Cell Carcinoma Found Inline with the Fields of Repeat Stereotactic Radiosurgery for Recurrent Trigeminal Neuralgia

**DOI:** 10.7759/cureus.2054

**Published:** 2018-01-12

**Authors:** Aldo Berti, Michelle Granville, Robert E Jacobson

**Affiliations:** 1 Miami Neurosurgical Center, University of Miami Hospital

**Keywords:** stereotactic neurosurgery, invasive squamous cell carcinoma, cyberknife radiosurgery, trigeminal neuralgia, gamma knife surgery, delayed effects of radiation, radiation induced secondary tumors, recurrent trigeminal neuralgia, repeat stereotatic radiosurgery

## Abstract

A case of an extremely healthy, active, 96-year-old patient, nonsmoker, is reviewed. He was initially treated for left V1, V2, and V3 trigeminal neuralgia in 2001, at age 80, with stereotactic radiosurgery (SRS) with a dose of 80 Gy to the left retrogasserian trigeminal nerve. He remained asymptomatic for nine years until his trigeminal pain recurred in 2010. He was first treated medically but was intolerant to increasing doses of carbamazepine and gabapentin. He underwent a second SRS in 2012 with a dose of 65.5 Gy to the same retrogasserian area of the trigeminal nerve, making the total cumulative dose 125.5 Gy. In late 2016, four years after the 2^nd^ SRS, he was found to have invasive keratinizing squamous cell carcinoma in the left posterior mandibular oral mucosa. Keratinizing squamous cell carcinoma is seen primarily in smokers or associated with the human papillomavirus, neither of which was found in this patient. A review of his two SRS plans shows that the left lower posterior mandibular area was clearly within the radiation fields for both SRS treatments. It is postulated that his cancer developed secondary to the long-term radiation effect with a very localized area being exposed twice to a focused, cumulative, high-dose radiation. There are individual reports in the literature of oral mucositis immediately after radiation for trigeminal neuralgia and the delayed development of malignant tumors, including glioblastoma found after SRS for acoustic neuromas, but there are no reports of delayed malignant tumors developing within the general radiation field. Using repeat SRS is an accepted treatment for recurrent trigeminal neuralgia, but physicians and patients should be aware of the potential effects of higher cumulative radiation effects within the treatment field when patients undergo repeat procedures.

## Introduction

Stereotactic radiosurgery (SRS) is a recognized treatment for trigeminal neuralgia after medication failure. The accepted treatment dose for the first SRS ranges between 70 and 90 Gy. The one year excellent to good response rate ranges from 75% to 86% for trigeminal neuralgia, however, long-term follow-up shows there is a gradual drop off in response rate with an increasing percentage of cases having recurrent pain [1]. Large studies of patients treated for trigeminal neuralgia with stereotactic radiosurgery show 60%-75% good results at three years, 46% to 51% at five years, and 22% to 27% at the 10-year follow-up [1-2]. As a result of this gradual recurrence of symptoms, many patients require repeat stereotactic radiosurgery. The results for repeat SRS range from 72% to 87% favorable results, very similar to the initial treatment, with the 2^nd^ treatment dose varying from 45 Gy to 90 Gy and, interestingly, there is less long-term decrease in good results than after the initial treatment [2-3]. The total dose exposure with two treatments has ranged from a low of 125 Gy to a high of 180 Gy with some centers advocating a higher second dose providing better long-lasting patient response due to more permanent change in the trigeminal nerve [2-3]. Side effects and complications are rare with the initial SRS treatment regardless of dose and range from 10% to 16% [1]. Immediate short-term complications are most commonly related to a neurologic change in the trigeminal nerve characterized by facial numbness, followed by increased facial pain, nausea, hearing loss, and urticaria [1]. Although numbness is listed as a complication, it is primarily a radiation effect on the trigeminal nerve. Other variables, such as length of the nerve treated and if the root entry zone is included in the radiation field, can also affect the incidence of numbness [1]. The incidence of facial numbness ranges from 6% to 11%, although most series relate better pain relief in patients that develop post-procedure numbness [1-3]. When patients undergo repeat SRS for recurrent trigeminal neuralgia, the incidence of sensory loss is slightly higher, ranging from 11% to 20% [2-3]. Secondary localized mucositis within four weeks after SRS treatment for trigeminal neuralgia was reported using a maximum dose of 76 Gy [4]. This was different from the generalized, usually self-limited, mucositis seen when whole brain or head and neck radiation treatments are first initiated [4]. Radiation for trigeminal neuralgia has rarely been associated with radiation necrosis as compared to whole brain radiotherapy. Radiation necrosis in the brain is volume related and although a very high dose is used with trigeminal neuralgia, it is concentrated on a very small volume measured in mm^3^ within the treatment field [5]. There are multiple case reports of long-term or delayed development of radiation-induced tumors in the brain, near areas where the stereotactic dose was administered [6-7]. However, this case is unusual with the occurrence of an oral squamous cell cancer developing 15 years after the original SRS treatment and four years after the second SRS but inline with the same target zone of both original treatments and inline with the anatomic pathway to approach the foramen ovale for trigeminal radiofrequency lesioning.

## Case presentation

**Figure 1:**
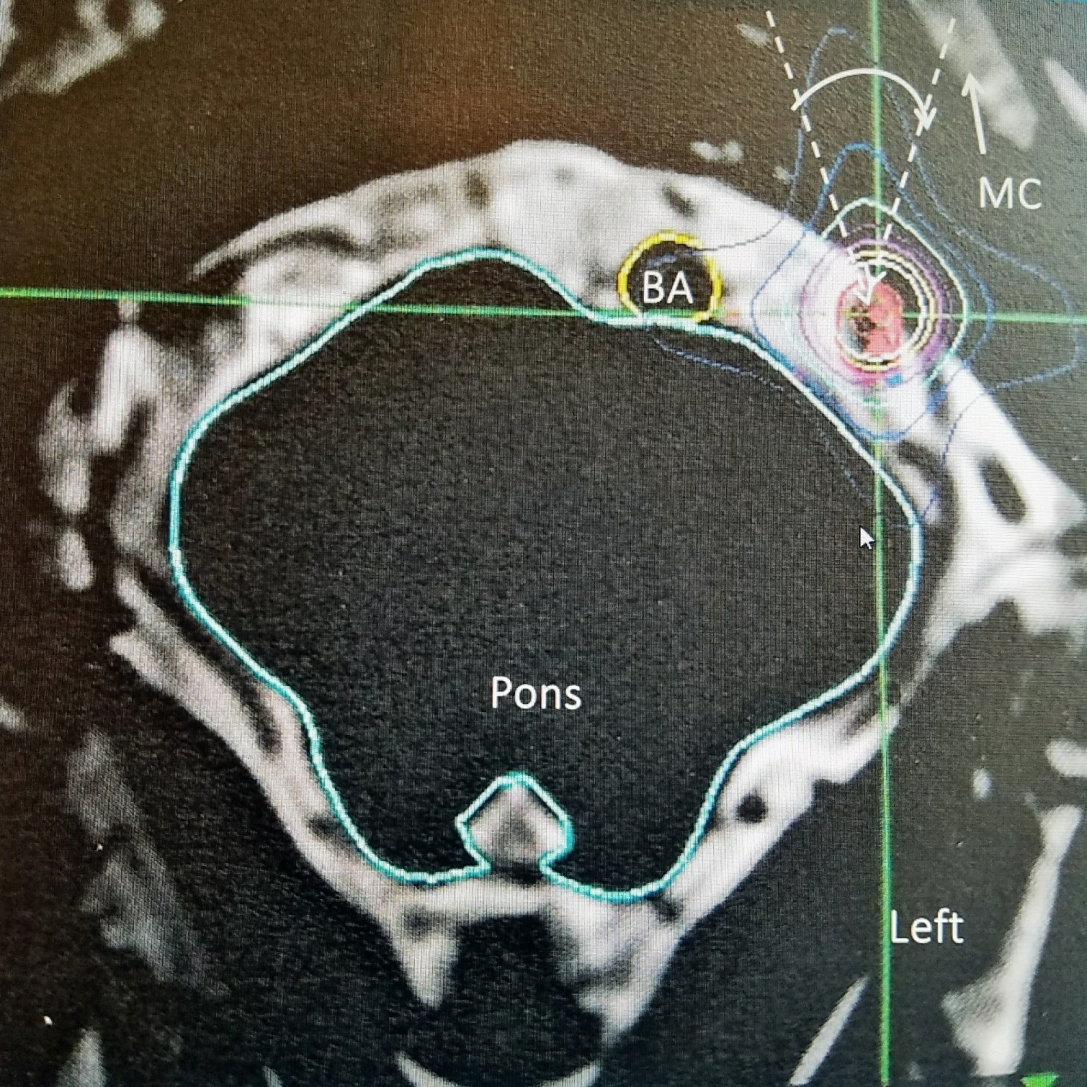
Axial T2 magnetic resonance image of stereotactic target to the left trigeminal nerve The 5 mm collimated target is aligned longitudinally along the axis of the trigeminal nerve. There is minimal edging along the brainstem and root entry zone. The basilar artery (BA) is identified as well as the mandibular condyle (MC). The dotted angle with the solid white arc is the spillover of beams toward the posterior left mandible where the oral carcinoma was found four years after the second stereotactic radiosurgery.

The patient is a 96-year-old, extremely healthy, and active male, nonsmoker, who was first treated with SRS for left facial trigeminal neuralgia in 2001. He initially presented 10 years prior to that with left cheek pain while eating, which spontaneously improved. He had a second crisis two years later and was managed medically with carbamazepine and gabapentin for many years. However, eight years later, he began to experience an 'electric shock' to his left cheek when shaving, talking, or eating. He was then offered SRS. This treatment was with the GammaKnife (GK) (Elekta, Stockholm, Sweden) using 80 Gy targeting 4 mm of the left retrogasserian trigeminal nerve. The brainstem dose was 98 cGy at the left trigeminal root entry zone. The patient's trigeminal neuralgia symptoms resolved, and he was placed on a maintenance dose of carbamazepine and gabapentin. After failing conservative treatment for a second time and with increasing pain in 2012, 11 years after his first SRS, he underwent a second stereotactic radiosurgery with the CyberKnife (CK) (Accuray Inc, Sunnyvale California) with 6,550 cGy utilizing one 5.0 mm collimator with a total of 104 beams employed to the same left trigeminal nerve. The brainstem dose was 84 cGy. He received a combined total dose of 145.5 Gy with both SRS treatments (Figure 1).

**Figure 2:**
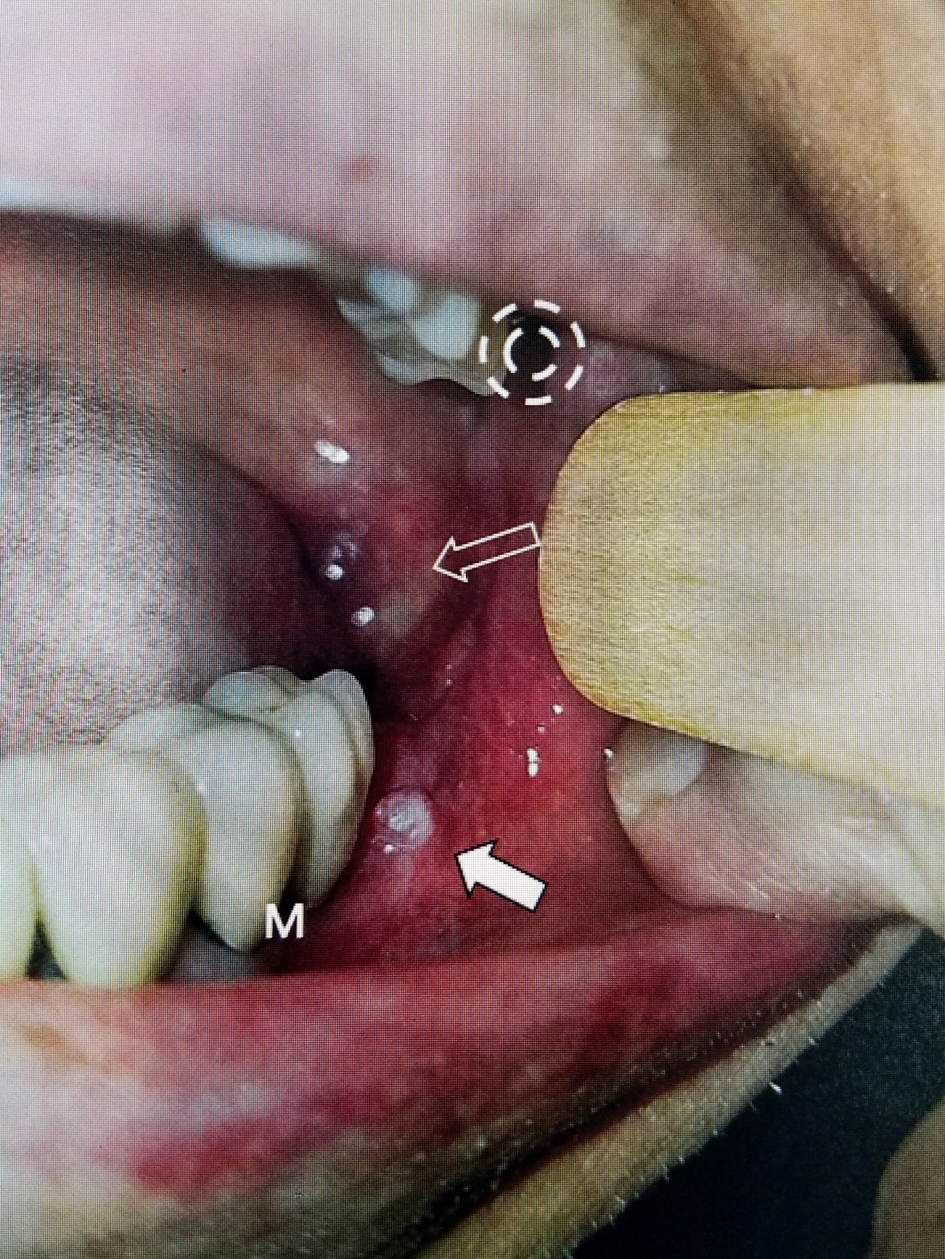
Photograph of the left posterior oral cavity showing a papilloma and tumor The solid white arrow shows the papilloma just lateral to the last molar. Above it is a second lesion in the posterior pharyngeal wall (open white arrow). Open white arrow shows the invasive squamous cell tumor extending posteriorly from the papilloma. The dashed circle shows the normal entry point for Gasserian ganglion injection and radiofrequency procedures in the retropharyngeal space near the pterygoid plate. The lesions are in direct alignment with the well-established trajectory for approaching the trigeminal nerve within Meckel's cave.

From 2012 to 2017, except for minor episodes of V1 and V2 pain, he remained pain-free and was managed with carbamazepine and gabapentin. Nonetheless, in 2017, he noticed a growth in the left mouth and posterior mandible (Figure 2).

**Figure 3:**
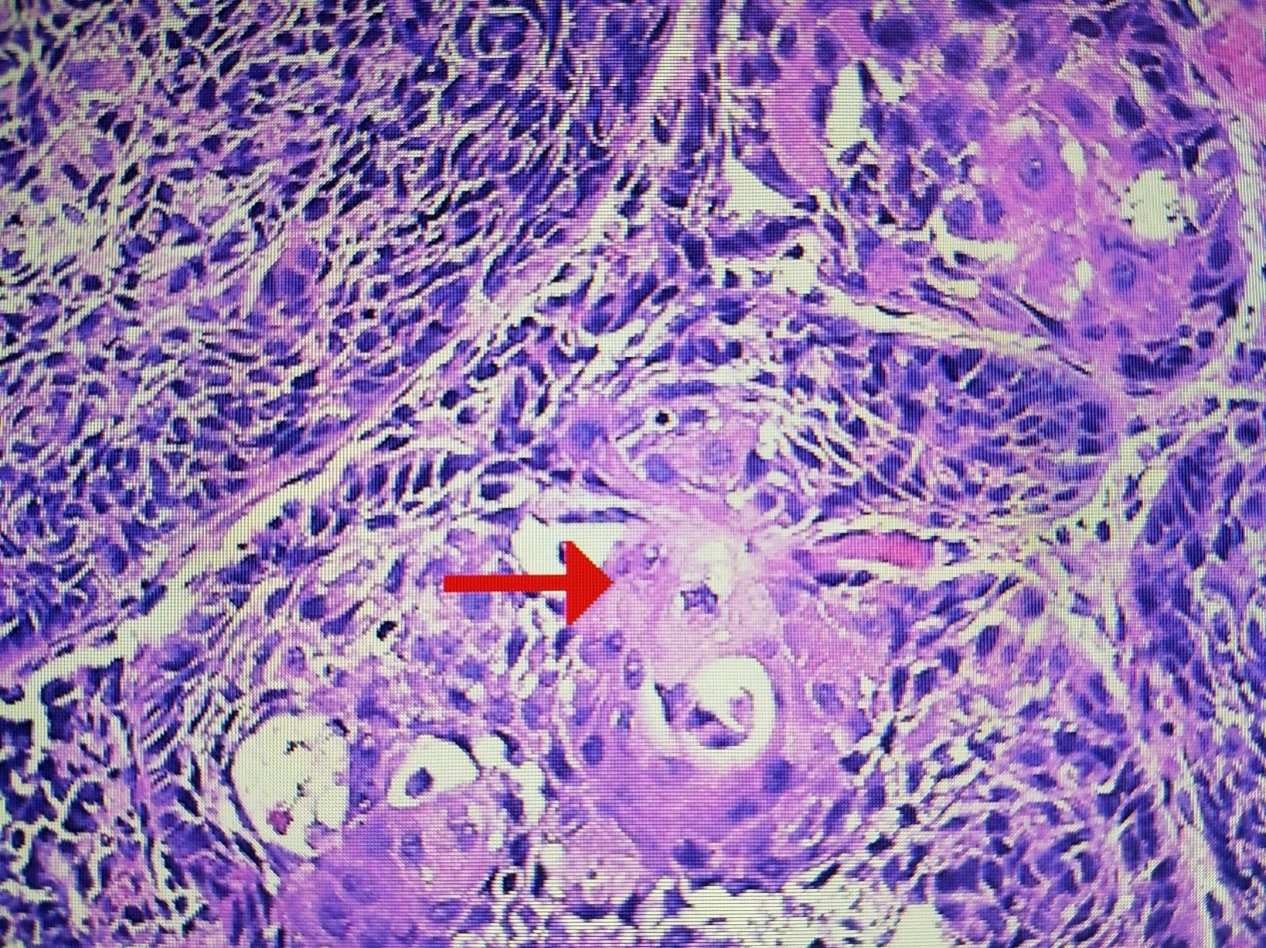
Pathological specimen for oral mucosa papilloma The arrow shows typical keratin formation around clusters of mitotic cells with squamous carcinoma. These are named keratin 'pearls.' There were no pathologic findings typical of human papillomavirus (HPV).

Upon further investigation with a positron emission tomography (PET) scan and biopsy, he was diagnosed with an invasive squamous cell carcinoma, keratinizing type. Testing for human papillomavirus (HPV) was found to be negative (Figure 3).

The oral carcinoma was then treated with intensity modulated radiation therapy (IMRT) using 6600 cGy. Follow-up has shown resolution of the cancerous area in the oral mucosa.

## Discussion

**Figure 4:**
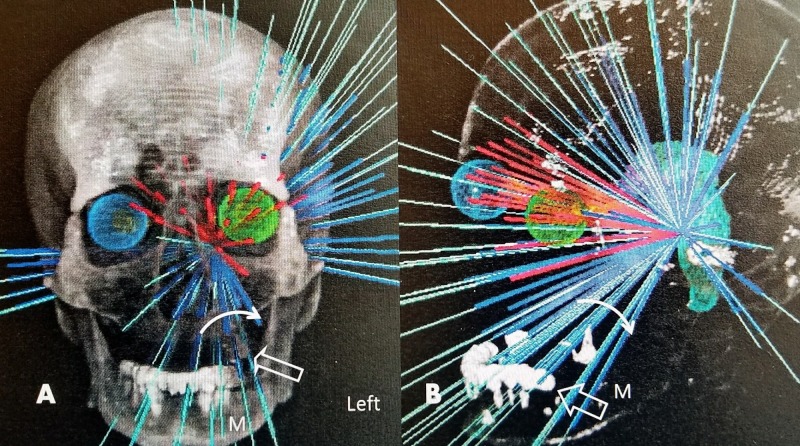
Linear distribution of the various CyberKnife beams for the second stereotactic treatment A: AP view showing the radiation beam array on the left targeting the trigeminal nerve. The area of the oral carcinoma is represented by the open white arrow. The solid arc shows the distribution of the separate radiation beams that passed through the left posterior mandibular (M) area. The beams are composed of both beams entering inferiorly and beams passing through the trigeminal ganglion from above. B: Lateral view showing the distribution and confluence of beams on the trigeminal nerve. The solid arrow arc shows the beams passing toward the left posterior mandible (M). The open white arrow is the area of the mucosal carcinoma.

Trigeminal neuralgia is a chronic disease that develops a progressively higher incidence of pain recurrence with long-term follow-up. These recurrences of pain often require further treatment, which includes a second or even third stereotactic radiosurgery (SRS) treatment, microvascular decompression, or radiofrequency rhizotomy of the trigeminal ganglion [1-3]. Repeat SRS is frequently the patient preference since it has good results and is the least invasive procedure. In large series of repeat SRS, the results are equal or better than initial treatment with an increased risk of facial numbness and minimal complications [2-3].This case raises two distinct but related issues. The first is understanding the range of doses used for repeat SRS treatment in patients with recurrent trigeminal neuralgia. It is clear that repeat SRS is effective, but there is debate regarding dosage [2-3]. The more experienced centers have been using a higher dosage with a maximum of 90 Gy, feeling that the resultant increased percentage of facial numbness actually accounts for longer and better results [3}. As a result, the range of total dosage reported varies from 140 to 180 Gy. Since radiosurgery to the trigeminal nerve and retrogasserian area involves such a small volume, this was felt to be within the radiation-safe zone [2]. Regardless of whether the isocentric GK or nonisocentric CK is used, the concept of stereotactic radiosurgery is to focus the beams on a central point, providing maximum radiation dose while minimizing the dose to surrounding tissue. However, with either system, the beams do not stop at the isocenter but proceed through the body, diverting outward, so there is radiation exposure to other tissues in line with the radiation beams but distant from the target. This can lead to secondary radiation effects in other areas within the path of the radiation beams, such as skull bone, skull base, brain, and oropharyngeal mucosa. This accounted for the case report of acute mucositis after initial CK treatment with a dose of cGy 7594 (or 76 Gy) [4]. Specifically, in this reported case, the mucositis was found in a direct linear path to the treatment beams used for targeting the trigeminal nerve and involved the upper lip, hard palate, and tongue. The beam array path was reviewed in detail, and it was found there were a number of overlapping beams and nodes (a point in the radiation treatment plan) where several beams emanate from the same fixed point) that could have been blocked, which directly affected the oral mucosa. Since radiation-induced mucositis is common but usually very generalized in the mouth and found at the start of radiation treatment, they hypothesized that the possibility of radiation hotspots and nodes in the nonisocenter array from the CK was a possible cause of the delayed onset and very localized mucositis [4]. In our case, the patient had two SRS treatments of 80 Gy and 65.6 Gy to the same overlapping area for a total of 145.5 Gy, 11 years apart. The initial SRS using the isocentric GK and the second using the nonisocentric CK. The first plan was no longer available, but the second stereotactic plan and stereotactic field array were reviewed retrospectively, after the discovery of the oral carcinoma. It is apparent that there were a number of beams passing through the involved oral mucosal and posterior pharyngeal wall that were in direct alignment with the location of the subsequently developed oral squamous cell carcinoma. Anatomically, this was in alignment with the path used for percutaneously accessing the foramen ovale to perform trigeminal rhizotomy (Figure 4).

This raises the question of radiation dose effect and the development of secondary malignant tumors. The concept of secondary radiation-induced malignant tumors was established by Cahan in 1948 with the very earliest experiences with radiation therapy. Cahan reported the delayed development of sarcoma in bones within the field of radiation. He described three tenants for radiation-induced tumors, first, a significant delay from radiation to tumor onset; second, the development of the malignant change within the field of radiation; and third, the tumor needed to be distant from the original tumor site and directly treated area [6]. There are reports of secondary malignant tumors in the path using both whole brain radiation as well as focused SRS, whether it is stereotactic radiosurgery or intensity modulated radiation therapy (IMRT). With brain SRS, there are reports of multiple cases of glioblastoma multiforme in the surrounding brain when treatment was for benign acoustic neuromas and the development of osteonecrosis and osteosarcoma in the skull bone within the radiation field after radiation treatment for a pineoblastoma [7-8]. More on point to this case, studies of the physics of different radiation systems, including both GK and CK, have shown calculating and measuring the actual radiation dose outside the target field can be inaccurate and often underestimates the peripheral dose. Considering that the radiation dose in stereotactic radiosurgery when treating trigeminal neuralgia is high, ranging between 70 and 90 Gy in a single dose, this observation is important [9]. There is also known to be a long latency period in radiation-induced effects. In studies of tumors developing after childhood whole brain and axis radiation for childhood leukemia, glial tumors are more commonly found in the first five years after whole brain radiation, while radiation-induced meningiomas, often multiple, are typically found after a very latent interval of 20 or more years. A pathologic study found that these meningiomas were atypical, very cellular, with aberrant mitotic changes showing malignant tendencies. These radiation-induced meningiomas were found to occur after both high- and low-dose cranial radiation therapy. Multiple follow-up studies showed a risk of both continued development of tumors and recurrence after resection. The incidence of these radiation-induced tumors continued to steadily increase over the years and did not plateau [10]. This indicates that radiation induces a permanent change in cellular kinetics, leading to recurrent tumor growth.

This case is cautionary since the patient is very elderly but extremely healthy, never smoked, was negative for HPV, and has a 16 plus year follow-up after the original CK radiosurgery for trigeminal neuralgia. There is no analysis in any of the reports, apart from those regarding post-irradiation tumors after childhood radiation, of the role age plays in this process [6-10]. As increasing numbers of radiosurgical procedures are performed with different equipment, it is important for physicians and patients to understand that although very minimal, there is a possible risk of malignant tumor development related to stereotactic radiosurgery, no matter how focused and small the target is perceived to be.

## Conclusions

Stereotactic radiosurgery has a very low rate of local and systemic complications. Repeat treatment does have the risk of secondary effects to the surrounding brain tissue. There are very few reports of extra-axial secondary effects from this type of focused radiation, but this report highlights that attention must be given to the entire radiation field, no matter how small and focused, such as in the treatment of trigeminal neuralgia. The use of high repeat doses of 70 to 90 Gy for second treatments of benign recurrent trigeminal neuralgia, adding to the original radiosurgical treatment, is not without potential risk for the development of delayed secondary radiation effects. This must be considered, especially in patients with long treatment follow-ups like this case, since secondary radiation effects can evolve after a long latency period.
